# The effect of deep extubation on the emergence agitation in pediatric patients undergoing obstructive sleep apnea syndrome surgery: a randomized controlled study

**DOI:** 10.1038/s41598-026-46227-0

**Published:** 2026-04-04

**Authors:** Yuanyuan Wan, Yun Shi, Xuan Wang, Jian Guo

**Affiliations:** Department of Anesthesiology, Children’s Hospital of Fudan University, Shanghai, People’s Republic of China

**Keywords:** Emergence agitation, Children, Obstructive sleep apnea syndrome, Deep extubation, Dexmedetomidine, Sevoflurane, Diseases, Health care, Medical research

## Abstract

Emergence agitation (EA) is a common complication during recovery from pediatric anesthesia, particularly in children with obstructive sleep apnea syndrome (OSAS) undergoing tonsillectomy and adenoidectomy (T&A). This study aimed to compare the effects of dexmedetomidine-assisted deep extubation and awake extubation strategies on the incidence of EA in this high-risk population. In this prospective randomized trial, 100 children aged 3–10 years scheduled for T&A were allocated to either a dexmedetomidine-assisted deep extubation group (*n* = 50) or an awake extubation group (*n* = 50). The primary outcome was the incidence of EA, assessed using the Pediatric Anesthesia Emergence Delirium (PAED) scale with a diagnostic threshold > 10. Secondary outcomes included extubation time and post-anesthesia care unit (PACU) length of stay. The dexmedetomidine-assisted deep extubation group demonstrated a significantly lower incidence of EA compared to the awake extubation group (21.3% vs. 54%; *p* = 0.002; relative risk [95% confidence interval] = 0.394 [0.215–0.722]; NNT [number needed to treat] = 3.06). Extubation time was also significantly shorter in the deep extubation group (5.5 ± 2.02 vs. 11.7 ± 2.39 min; *p* < 0.001). No significant difference was observed in PACU length of stay between the two groups (60.4 ± 2.92 vs. 60.6 ± 3.14 min; *p* = 0.78). In this single-center randomized controlled trial, dexmedetomidine-assisted deep extubation was associated with a reduced incidence of EA and a shorter extubation time, without prolonging PACU stay or increasing observed adverse events.

**Trial registration:** Chictr.org.cn; Identifier: ChiCTR2300075947; registered 20 September 2023.

## Introduction

Emergence agitation (EA) is common in children during recovery from general anesthesia, characterized by moaning, restlessness, disorientation, thrashing and non-purposeful movement^1^. Although typically self-limiting, EA can lead to serious complications and safety risks, including patient falls (e.g., from bed), surgical site bleeding, accidental dislodgement of drains or catheters, and a significant increase in nursing workload in the post-anesthesia care unit (PACU)^2,3^.

The etiology of EA after general anesthesia remains unclear, the potential causes include rapid emergence, sevoflurane anesthesia, pain, younger age, and specific surgical procedures^4,5^. Reported incidence rates vary widely, from 0.25% to 90.5%, and EA is notably common after tonsillectomy and adenoidectomy (T&A) in children with obstructive sleep apnea syndrome (OSAS)^6,7^. While various pharmacological interventions have been employed to prevent or alleviate EA symptoms, it remains a significant challenge to optimal patient care. These medications may cause adverse effects such as respiratory depression, prolonged recovery time, and delayed discharge from the PACU^8^.

Current evidence remains limited regarding the influence of extubation techniques on EA incidence following T&A. To address this gap, we conducted a prospective randomized controlled trial comparing the efficacy and safety of dexmedetomidine-assisted deep extubation versus awake extubation for reducing EA in pediatric OSAS patients after T&A.

## Materials and methods

### Study design and setting

This study adhered to the Good Clinical Practice guidelines and was conducted in accordance with the ethical principles outlined in the Declaration of Helsinki. The prospective randomized controlled trial was approved by the Ethics Committee of the Children’s Hospital of Fudan University (Approval No.202314) and registered with the Chinese Clinical Trial Registry (ChiCTR2300075947; Registration Date: 20/09/2023). Written informed consent from the parent or legal guardian was obtained for all patients prior to study inclusion.

**Fig. 1:**
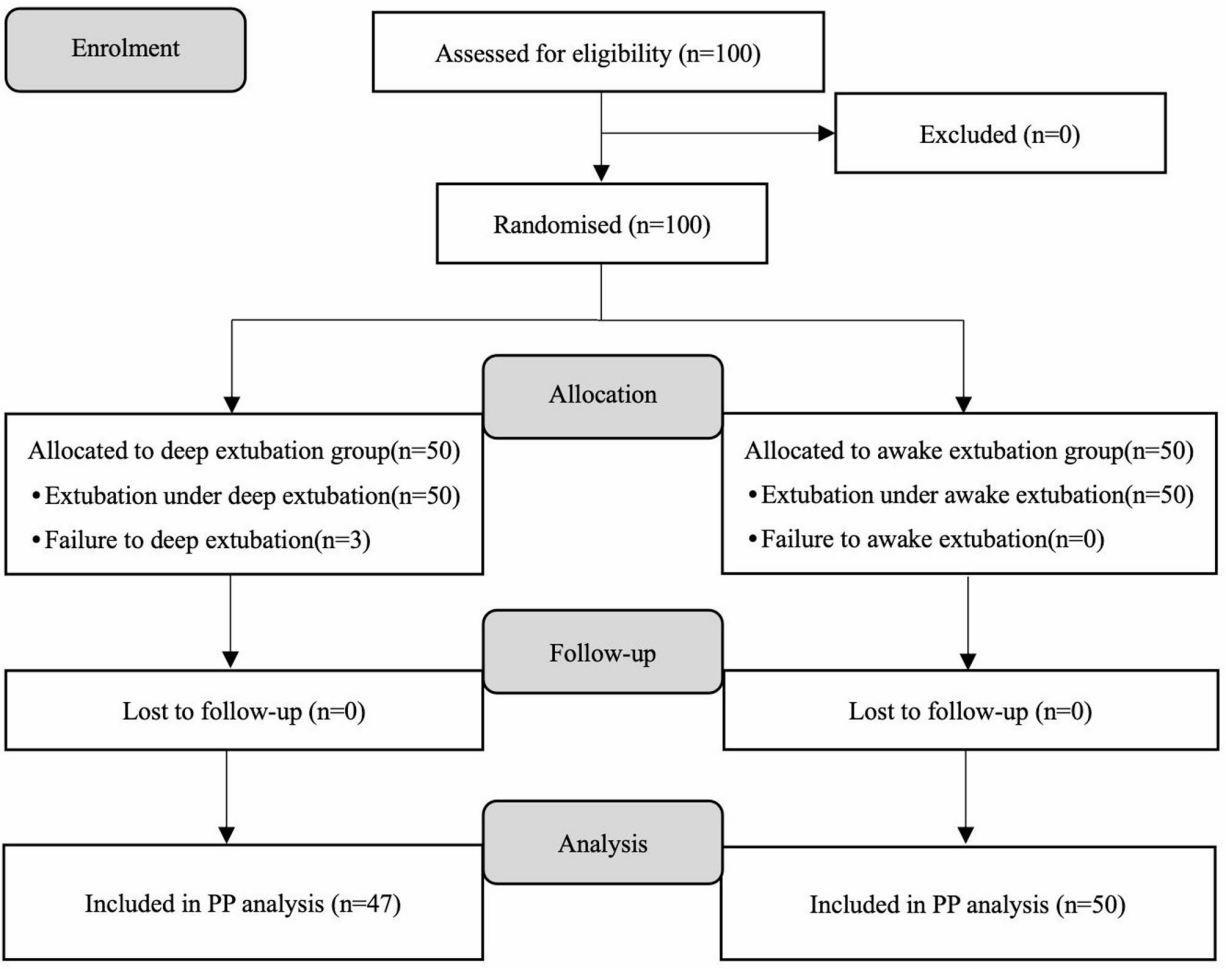
Consolidated standards of reporting trials (CONSORT) flowchart.

The study cohort comprised pediatric patients aged 3–10 years with American Society of Anesthesiologists (ASA) physical status I or II who were scheduled for elective T&A. Exclusion criteria included: active upper respiratory tract infection (within 2 weeks), neurological impairments, documented asthma, anthropometric obesity (BMI exceeding the 95th percentile for age-sex norms), congenital craniofacial abnormalities, gastroesophageal reflux and difficult airway. Patient recruitment and allocation followed CONSORT guidelines as detailed in Fig. 1.

### Randomization and intervention

Patients were randomly assigned in a 1:1 ratio to either the deep extubation group or the awake extubation group. An independent statistician generated the randomization sequence using block randomization with a block size of 4. To ensure allocation concealment, the group assignments were placed in sequentially numbered, opaque, sealed envelopes. At the end of surgery, an independent anesthesiologist opened the next sequentially numbered envelope to reveal the group assignment.

### Blinding

Due to the nature of the intervention, the anesthesiologist performing the intraoperative care and extubation could not be blinded to the group assignment. However, to minimize assessment bias, all postoperative evaluations in the PACU were conducted by a separate anesthesiologist and a research nurse who were blinded to the group allocation. These blinded assessors were responsible for collecting all outcome data, including the primary endpoint of EA.

### Procedure

No preoperative sedatives were administered prior to transfer to the operating room. Upon arrival, comprehensive physiological monitoring was established, including electrocardiography (ECG), non-invasive blood pressure (NIBP), pulse oximetry (SpO_2_). Anesthesia was induced via inhalation of 8% sevoflurane with an oxygen flow of 4 L/min. Tracheal intubation was facilitated with intravenous propofol (2 mg/kg). Prior to intubation, each child received atropine 0.01 mg/kg, dexamethasone 0.25 mg/kg (up to 10 mg), hydromorphone 7 µg/kg and propacetamol 30 mg/kg (maximum dose 1 g). Neuromuscular blocking agents were not administered during the procedure to facilitate the maintenance of spontaneous breathing and to allow for immediate assessment of respiratory adequacy at the time of extubation. Anesthesia maintenance utilized a low-oxygen mixture (Fio_2_<0.30) combining sevoflurane with medical air/oxygen. Intravenous ondansetron (0.1 mg/kg, maximum 4 mg) was administered during the final surgical phase. Intraoperative hemodynamic activation, manifested as heart rate or blood pressure elevations exceeding 20% of preoperative baselines, was controlled through incremental augmentation of inhaled anesthetic concentrations under continuous vital sign monitoring.

At the end of surgery, tracheal extubation was performed according to the group assignment. All children received 100% oxygen prior to extubation. In the awake extubation group, all anesthetic drugs were discontinued and tracheal extubation undertaken when the child had demonstrated facial grimacing, adequate tidal volumes(≥ 5 ml/kg), purposeful movements and eye opening. In the dexmedetomidine-assisted deep extubation group, spontaneous breathing was resumed with inhaled sevoflurane greater than 1 minimum alveolar concentration (end-tidal concentration of sevoflurane is about 1.7%)^9^ and a dexmedetomidine bolus (0.5 µg/kg) was administered intravenously over 10 s using a 2 µg/mL concentration. Thereafter, the oropharynx was gently suctioned, and the endotracheal tube cuff was deflated, if spontaneous breathing was maintained or restored following a transient apnea (< 5 s), indicating readiness for deep extubation. Once adequate spontaneous respiratory༈5 ml/kg༉was confirmed, the endotracheal tube was removed quickly and gently. Sevoflurane was discontinued after extubation, and the patency of the upper airway was confirmed by the presence of stable, unobstructed spontaneous breathing with adequate chest rise and capnography waveform. Once the airway was confirmed to be patent and stable, the patient was transferred to the PACU and placed in a lateral position until consciousness recovered. All children were continuously monitored. Recording anesthesia time (from the start of anesthesia induction to the discontinuation of anesthetic agents at the conclusion of surgery) and surgery time (from the start of the surgical dissection to the completion of the procedure with confirmed hemostasis).

### Outcome assessments

The primary outcome was the incidence of EA. EA was assessed by a nurse blinded to group allocation using the Pediatric Anesthesia Emergence Delirium (PAED) scale at 10-minute intervals throughout the entire PACU stay, starting from arrival until discharge. Given that the deep extubation group emerges from anesthesia in the PACU (after transfer), while the awake extubation group emerges prior to PACU arrival, the PACU arrival time point represents the earliest feasible time for standardized, environment-controlled assessment in both groups. This approach captures the emergence period in the setting where clinical management and safety events occur. PAED scores > 10 indicated EA. EA was defined as the occurrence of any single PAED score > 10 at any of these assessment time points. The management of EA and postoperative pain in the PACU followed a pre-defined institutional protocol. For children with a PAED score > 10 exhibiting agitation, intravenous propofol (1–2 mg/kg) was administered. This dose was sufficient to terminate the agitated behavior, consistent with its rapid hypnotic effect. Subsequent assessment for pain was performed after the child regained calmness. If distress persisted or pain was suspected after recovery from sedation, supplemental fentanyl (0.5 µg/kg) was given. During this period, patients’ vital signs were closely monitored, and all adverse events were recorded. Respiratory adverse events were prospectively defined according to the following criteria: Laryngospasm: Partial or complete airway obstruction requiring positive pressure ventilation and/or administration of succinylcholine. Hypoxemia: Peripheral oxygen saturation (SpO_2_) < 90% lasting for more than 30 s. Apnea: Cessation of breathing for > 20 s. Airway obstruction: Stridor or evidence of upper airway obstruction requiring manual intervention (chin lift, jaw thrust) or insertion of an oral or nasal airway. Monitoring throughout the perioperative period included continuous electrocardiography (ECG), non-invasive blood pressure (NIBP), and pulse oximetry (SpO_2_). Capnography was used in the operating room and upon arrival in the post-anesthesia care unit (PACU) until the patient was fully awake and had stable respiratory function. Respiratory rate was measured continuously by the patient monitor and recorded every 10 minutes by the attending nurse or anesthesiologist. We also recorded other adverse events in the PACU, including cough and postoperative nausea and vomiting (PONV). Cough results were assessed using the modified four-point Minogue scale. Grade 1: no cough; grade 2: (mild) once or twice; grade 3: (moderate) fewer than four, or lasting less than 5 s; and grade 4: (severe) at least four coughs, or overall coughing duration being more than 5 s^10^. PONV: the occurrence of at least one episode of vomiting in the PACU. Record the emergence time for children in the deep extubation group (defined as the time from arrival in the PACU to the return of consciousness). The secondary outcomes included extubation time (time from the end of surgery to tracheal extubation) and PACU length of stay (from the time of PACU admission until the patient’s discharge from the PACU). The primary outcome and core secondary outcomes were pre-specified in the registered study protocol (ChiCTR2300075947). Exploratory secondary outcomes (PAED score as a continuous variable, cough incidence, rescue medication use, and perioperative adverse events) were added as part of standard clinical safety monitoring and granular EA severity assessment. Patients were discharged from the PACU to the general ward when they met all of the following criteria: (1) a modified Aldrete score of ≥ 9; (2) stable vital signs for at least 1 h; (3) no active bleeding; (4) minimal or no pain (FLACC scale < 4); and (5) absence of significant nausea or vomiting.

### Sample size

Based on our pilot observations and consistent with existing literature^11,12^, the incidence of EA was estimated to be 60%. If we expected a 50% reduction in the incidence of EA with deep extubation and 10% of patients dropped out of the study, to detect the difference at 80% power with a two-sided alpha of 0.05, 50 patients in each group were required.

### Statistical analysis

Statistical analyses were performed using SPSS 29.0 (Statistical Package for the Social Sciences, IBM SPSS Inc., Chicago, IL, USA). An independent sample t-test or the Mann-Whitney U test was used to compare continuous variables between the two groups, depending on the normality distribution of data, using the Shapiro-Wilk test. Data are presented as mean ± standard deviation (SD) for continuous variables with normal distribution, median [range] for non-normally distributed variables, and number (%) for categorical variables. Comparisons between groups were made using Student’s t-test for normally distributed continuous variables, and the chi-square test or Fisher’s exact test for categorical variables, as appropriate. To evaluate the potential bias introduced by rescue propofol administration, a sensitivity analysis was performed for the primary outcome. In this analysis, patient data were censored at the time of propofol administration; only PAED scores obtained before rescue propofol were considered, and patients who received propofol were still classified as having EA if their pre-rescue PAED score exceeded 10. Given the significant difference in anesthesia time observed between the two groups, an Analysis of Covariance (ANCOVA) was performed to assess the effect of group allocation on PAED scores, with anesthesia time entered as a covariate. This analysis was conducted to determine whether the group difference in PAED scores remained significant after adjusting for the potential confounding effect of anesthesia duration. A *p* value less than 0.05 was considered statistically significant.

## Results

A total of 100 children underwent elective T&A between October 2023 and January 2024 were included in this study. In the dexmedetomidine-assisted deep extubation group, three patients were excluded: two due to movement before the end of surgery requiring additional anesthetic, and one due to excessive airway secretions deemed unsuitable by the attending anesthesiologist. No patients were excluded based on preoperative assessment of difficult airway or severe gastroesophageal reflux disease. Therefore, 47 patients in the dexmedetomidine-assisted deep extubation group and 50 patients in the awake extubation group were included in the statistical analysis (Fig. 1). Patient characteristics, anesthetic and surgical information of each group are presented in Table 1. Both the dexmedetomidine-assisted deep extubation and awake extubation group were similar with respect to age, age proportions, sex, weight and surgery time.

**Table 1:** Patient characteristics, anesthetic and surgical information.

	Deep extubation group (*n* = 47)	Awake extubation group (*n* = 50)	*p* value
Age (years)	5.13 ± 1.53	5.18 ± 1.61	0.87
Age proportions [n (%)]			
3–<5 years	17 (36.2%)	20 (40%)	0.70
5–10 years	30 (63.8%)	30 (60%)	
Sex (male/female)	34/13	29/21	0.14
Weight (kg)	21.1 ± 5.76	23.2 ± 9.29	0.19
Anesthesia time (min)	29.8 ± 6.21	38.4 ± 6.98	< 0.001
Emergence time (min)	20.7 ± 4.76	0	< 0.001
Surgery time (min)	20.6 ± 6.50	21 ± 7.27	0.52

Data expressed as mean ± SD or number (%).

### Primary outcomes

The incidence of EA was 21.3% (10/47) in the dexmedetomidine-assisted deep extubation group and 54.0% (27/50) in the awake extubation group, a significant difference in incidence was found between the two groups (*p* = 0.002; relative risk [95% confidence interval] = 0.394 [0.215–0.722]; NNT [number needed to treat] = 3.06) (Table 2). A sensitivity analysis, in which patient data were censored at the time of rescue propofol administration, confirmed the primary finding. The incidence of EA remained significantly lower in the dexmedetomidine-assisted deep extubation group compared to the awake extubation group (21.3% vs. 54.0%, *p* = 0.002). This indicates that the administration of propofol did not bias the assessment of the primary outcome. Moreover, the PAED score of the dexmedetomidine-assisted deep extubation group was significantly lower than that of the awake extubation group [5.85 ± 3.80 vs. 8.18 ± 5.10; *p* < 0.001].

**Table 2:** Outcomes and interventions.

	Deep extubation group (*n* = 47)	Awake extubation group (*n* = 50)	*p* value	RR (95%CI)
Incidence of EA [*n* (%)]	10 (21.3%)	27 (54%)	0.002	0.394 [0.215–0.722]
PAED score	5.85 ± 3.80	8.18 ± 5.10	< 0.001	–
Extubation time (min)	5.5 ± 2.02	11.7 ± 2.39	< 0.001	–
PACU length of stay (min)	60.4 ± 2.92	60.6 ± 3.14	0.78	–
Incidence of rescue propofol [n (%)]	6 (12.8%)	18 (66.7%)	0.008	0.355 [0.154–0.816]
Incidence of rescue fentanyl [n (%)]	2 (4.3%)	3 (6%)	1.000	0.709 [0.124–4.060]
Incidence of protective restraint [n (%)]	2 (4.3%)	6 (12%)	0.273	0.355 [0.076–1.659]
Incidence of parent presence [n (%)]	1 (2.1%)	3 (6%)	0.384	0.355 [0.038–3.291]

Data are described as mean ± SD or number (%). EA, emergence agitation; PAED, Pediatric Anesthesia Emergence Delirium scale; PACU, post anesthesia care unit.

To address the inherent difference in emergence and extubation times between groups, which could theoretically shift the observation window, a post-hoc sensitivity analysis was performed. We compared the incidence of EA within the first 30 min after PACU admission, a period that encompasses the peak emergence phase for both groups. The results of this analysis were consistent with the primary analysis, showing a significantly lower incidence of EA in the deep extubation group (21.3% [10/47] vs. 52.0% [26/50]; *p* = 0.003), confirming that the timing of the observation window did not confound the primary outcome.

### Secondary outcomes

There were significant differences between the groups for extubation time [5.5 ± 2.02 vs. 11.7 ± 2.39; *p* < 0.001] and anesthesia time [29.8 ± 6.21 vs. 38.4 ± 6.98; *p* < 0.001]. In the dexmedetomidine-assisted deep extubation group, 6 out of the 10 patients who developed EA (12.8% of the total group) received rescue propofol, whereas in the awake extubation group, 18 out of the 27 patients with EA (66.7% of the EA patients in that group) received propofol (*p* = 0.008). There was no significant difference in the use of rescue fentanyl between groups (4.3% vs. 6.0%, *p* = 1.000). There was no difference between the groups for PACU length of stay (60.4 ± 2.92 vs. 60.6 ± 3.14; *p* = 0.78) (Table 2).

As shown in Table 1, anesthesia time was significantly longer in the awake extubation group compared to the deep extubation group (38.4 ± 6.98 vs. 29.8 ± 6.21 min, *p* < 0.001). To evaluate whether this difference confounded the primary outcome, an Analysis of Covariance (ANCOVA) was performed with PAED score as the dependent variable, group allocation as the fixed factor, and anesthesia time as a covariate. The results showed that the group effect remained significant after controlling for anesthesia time [F (1, 94) = 9.24, *p* = 0.003], indicating that the lower PAED scores in the deep extubation group were independent of the difference in anesthesia duration.

### Adverse events

The incidence of cough was recorded as an adverse event and analyzed as an exploratory, post-hoc outcome. In the dexmedetomidine-assisted deep extubation group, the incidence of coughing was lower (8.5%% vs. 32%; *p* = 0.004) (Table 3). One patient dislodged the intravenous catheter during an episode of EA in the awake extubation group. No adverse events such as apnea, hypoxemia (SpO_2_<90%), laryngospasm, drowsiness or bradycardia were noted. No significant difference was observed between the two groups in the incidence of PONV (8.5% vs. 14%; *p* = 0.394) (Table 3). No adverse events related to dexmedetomidine, such as bradycardia (heart rate < 60 bpm or < 20% below the age-adjusted normal range) or hypotension, were observed in the deep extubation group.

**Table 3:** Adverse events in PACU.

	Deep extubation group (*n* = 47)	Awake extubation group (*n* = 50)	*p* value
Predefined respiratory adverse events			
Apnea	0	0	-
Hypoxemia (SpO_2_<90%)	0	0	-
Laryngospasm	0	0	-
Airway obstruction requiring intervention	0	0	-
Other adverse events			
Cough	4 (8.5%)	16 (32%)	0.004
No cough	43	34	0.004
Mild cough	2	4	0.678
Moderate cough	2	10	0.019
Severe cough	0	2	0.495
PONV	4 (8.5%)	7 (14%)	0.394

Data are described as number (%). PONV, postoperative nausea and vomiting.

## Discussion

The present study demonstrated that dexmedetomidine-assisted deep extubation was associated with a reduced incidence of EA and a shorter extubation time, without prolonging PACU stay or increasing observed adverse events.

EA is one of the most common complications following sevoflurane-based anesthesia in children. It may result in unnecessary harm to children, cause parental concern, and increase utilization of PACU resources. EA is a self-limiting phenomenon, which usually occurs within the first 30 min after anesthesia^2^. The 54% incidence observed in our awake extubation group aligns with reported rates for children undergoing T&A with sevoflurane^12–14^. The wide variability in reported EA incidence is attributable to multiple factors including medications, postoperative pain, patient age, and assessment tools.

Endotracheal extubation is a critical procedure during emergence from general anesthesia. Commonly, tracheal extubation is performed once patients are awake, with the return of both consciousness and protective airway reflexes. However, this awake extubation technique is frequently associated with a high incidence of EA, particularly in pediatric patients^15,16^, it can decrease the patients’ quality of recovery and cause severe complications and adverse events^2,3^. To prevent the unwanted side effect of awake extubation, extubation under deep anesthesia was advocated^17^. In our study, we utilized low dose dexmedetomidine (0.5 µg/kg) to facilitate smooth extubation under deep anesthesia which significantly reduced the incidence of EA in children during the recovery period. Dexmedetomidine was not administered in the awake extubation group, as previous evidence suggests it may be associated with prolonged extubation time^18^. Hauber et al. reported that using dexmedetomidine during deep anesthesia extubation also reduces the incidence of EA in pediatric patients^11^. But, In Lee’s study, the incidence of EA with deep extubation was quite similar to that of awake extubation^19^. This may be attributed to the following reasons: first, the different type of surgery, several studies have identified T&A as risk factor for EA in pediatric patients^20^; Second, dexmedetomidine, a meta-analysis show that, dexmedetomidine can reduce the incidence of EA in pediatric patients after sevoflurane anesthesia compared to placebo^21^, but, dexmedetomidine was administered in this study was primarily based on its pharmacological properties to attenuate airway reflexes and facilitate smoother extubation^22^; Third, assessment tools for EA, the assessment scales used in it differ from those employed in our study. Consequently, there may be discrepancies between the results of the two studies.

Propofol (1-2 mg/kg) was chosen as the first-line rescue for severe EA in OSAS children based on its rapid onset (30–60 s) and short duration of action (5–10 min)—critical for avoiding prolonged respiratory depression in a population with baseline airway obstruction. All propofol administrations were performed under continuous SpO_2_ and respiratory rate monitoring with 100% oxygen support, and no cases of hypoxemia (SpO_2_<90%) or respiratory depression were observed. The rapid redistribution of propofol in pediatric patients ensures sedative efficacy without prolonged recovery—consistent with our finding of no difference in PACU stay duration between groups.

Rather than focusing on pharmacological agents, the present study examined airway management techniques as the primary intervention. Rapid awakening by unfamiliar medical staff in unfamiliar environments has been described as a potential risk factor for EA^20^. The emergence time delayed in the deep extubation group may facilitate sevoflurane washout before the point of emergence, and not prolong the length of stay in the PACU. Additionally, deep extubation may mitigate emergence related psychological distress in pediatric patients, potentially preventing perioperative fear or anxiety associated with awake tracheal tube removal and with sudden exposure to an unfamiliar environment.

Postoperative pain is a major confounding factor for EA, yet it is difficult to measure accurately in children, especially when agitation is present. In our study, hydromorphone was administered intraoperatively at a relatively safe dose (7 µg/kg) for postoperative analgesia^23^. In our clinical experience, this dosage regimen demonstrates effective analgesic efficacy. We also have employed non-opioid analgesics (30 mg/kg propacetamol) as part of a multimodal analgesia regimen. In the PACU, propofol was administered as the primary intervention for EA. Supplemental analgesia was administered at the discretion of the anesthesiologist in the PACU following the children were full recovery of consciousness.

In the clinical setting of pediatric anesthesia, where high turnover rates are common, deep extubation may offer advantages. In our study, the dexmedetomidine-assisted deep extubation group significantly reduced extubation time (5.5 ± 2.02 vs. 11.7 ± 2.39 min), this may be due to awake extubation requires waiting for full return of consciousness and protective airway reflexes, while deep extubation is performed as soon as adequate spontaneous breathing is restored. It may also enhance the turnover rate of pediatric anesthesia. Additionally, the deep extubation group exhibited a markedly lower rate of coughing^24^, which may also be associated with the use of dexmedetomidine, a meta-analysis suggests that dexmedetomidine can reduce emergence coughing^10^. Furthermore, we did not demonstrate any other difference in the incidence of perioperative complication following awake or deep extubation, these results align with prior research findings^25,26^.

An important finding of this study is the absence of major respiratory adverse events, including laryngospasm, desaturation (SpO_2_ < 90%), and apnea, in both the dexmedetomidine-assisted deep extubation group and the awake extubation group. While this observation supports the safety of our deep extubation protocol in carefully selected children with OSAS undergoing tonsillectomy and adenoidectomy, it must be interpreted with appropriate caution. First, the study was powered for the primary outcome of EA incidence, not for rare respiratory complications. With approximately 100 patients, it is underpowered to detect differences in low-frequency events such as laryngospasm (estimated incidence < 5%). Thus, the absence of observed events does not demonstrate safety equivalence. Second, the favorable safety profile likely reflects institutional factors: all procedures were performed at a high-volume tertiary pediatric hospital by experienced anesthesiologists using a protocol that included dexmedetomidine (0.5 µg/kg), confirmation of adequate spontaneous breathing (≥ 5 mL/kg) before extubation, immediate post-extubation CPAP (5–10 mmHg), and continuous capnography monitoring. These factors limit generalizability to other settings with different patient populations, protocols, or expertise levels.

### Limitations

This study has several limitations. First, we did not use a validated pain scale to differentiate pain from emergence agitation (EA). Since pain is a major driver of postoperative agitation, some episodes classified as EA may have been pain-related, despite our multimodal analgesic regimen. Our protocol of administering propofol first for agitation, followed by pain assessment after the child calmed, is a pragmatic but imperfect approach to this challenge. Second, anesthesia time was significantly longer in the awake extubation group (38.4 vs. 29.8 min, *p* < 0.001), primarily due to the inherent waiting time required for awake extubation. Although prolonged sevoflurane exposure is a risk factor for EA, ANCOVA analysis confirmed that the group effect on PAED scores remained significant after adjusting for anesthesia time (*p* = 0.003). However, residual confounding cannot be completely excluded. Third, a key limitation of our study design is that we cannot differentiate the individual contributions of the deep extubation technique versus dexmedetomidine to the observed reduction in EA. The intervention was a bundled protocol (dexmedetomidine-assisted deep extubation). Therefore, whether the effect is primarily driven by the pharmacological action of dexmedetomidine, the avoidance of awake stimulation, or a synergistic interaction remains unknown. Future trials with factorial designs (e.g., deep extubation with/without dexmedetomidine, awake extubation with/without dexmedetomidine) are necessary to disentangle these effects. Fourth, as a single-center study conducted at a tertiary pediatric hospital by experienced anesthesiologists using a specific dexmedetomidine-assisted protocol, the findings may have limited generalizability to other institutions, patient populations, or deep extubation techniques. Finally, the study was not powered to evaluate safety outcomes. Although no major respiratory adverse events were observed, this should not be interpreted as proof of safety equivalence given the sample size. Larger multicenter trials are needed to confirm both efficacy and safety.

## Conclusion

In this single-center study, dexmedetomidine-assisted deep extubation was associated with a reduction in EA and faster extubation in children undergoing T&A, without significant increases in recovery time or observed respiratory complications. These findings suggest a potential benefit of this combined technique. However, given the sample size limitation and the single-center design, the results should be considered exploratory and warrant further investigation in larger, randomized trials to confirm efficacy and safety.

## Data Availability

The datasets used and analyzed in this study are available from the corresponding author upon reasonable request.

## Abbreviations

EA: Emergence agitation
OSAS: Obstructive sleep apnea syndrome
T&A: Tonsillectomy and adenoidectomy
PAED: Pediatric Anesthesia emergence delirium
PACU: Post-anesthesia care unit
ASA: American society of anesthesiologists classification
BMI: Body mass index
ECG: Electrocardiography
NIBP: Non-invasive blood pressure
SpO_2_: Pulse oximetry
NNT: Number needed to treat
